# Production of an Innovative, Surface Area‐Enhanced and Biodegradable Biofilm‐Generating Device by 3D Printing

**DOI:** 10.1002/elsc.202400046

**Published:** 2025-02-24

**Authors:** Atulona Datta, Rituparna Saha, Sovan Sahoo, Arup Ratan Roy, Shayontani Basu, Girish Mahajan, Subhash Chandra Panja, Joydeep Mukherjee

**Affiliations:** ^1^ School of Environmental Studies Jadavpur University Kolkata India; ^2^ Department of Mechanical Engineering Jadavpur University Kolkata India; ^3^ HiMedia Laboratories Pvt. Ltd. Thane (West) India

**Keywords:** Additive manufacture, Biofouling, Bioremediation, Confocal laser scanning microscopy, Medical implant

## Abstract

The enhanced surface cylindrical flask (ESCF) consists of an eight‐striped inner arrangement holding 16 standard microscopic slides placed inside a cylindrical vessel. The specially designed spatula‐accessible slides can be withdrawn from the vessel during cultivation without disturbing biofilm formation through an innovative window‐flap accessibility mechanism. The vessel and its accessories were three‐dimensional (3D) printed by applying a fused deposition modeling technique utilizing biodegradable polylactic acid. Biofilms of clinically relevant bacteria namely *Klebsiella pneumoniae*, *Pseudomonas aeruginosa*, *Staphylococcus aureus*, and *Escherichia coli* were successfully grown in the ESCF and observed through confocal laser scanning microscopy. Advantages of the device include an enhanced surface area for biofilm formation, ease of insertion and removal of microscopic slides, convenient fitting into standard rotary shaker platforms, creation of anoxic/microaerophilic environment inside the vessel as well as the feasibility of pH, dissolved gases, and metabolite measurements in the liquid surrounding the biofilm. The ESCF will find widespread application in medical, industrial, and environmental disciplines.

## 1 Introduction

Microbial biofilms are advantageous such as in wastewater treatment systems, but have adverse impacts when formed in industrial vessels and pipelines or on medical implants. Research on the formation and elimination of biofilms is important in medical, industrial and environmental disciplines. Novel methods have evolved, or have been adapted to biofilm studies for gaining advanced knowledge on biofilm composition, structure and physiology. Biofilm technology has progressed toward advancing design and production of biofilm‐cultivation devices that allow abundant biofilm formation as well as convenient determination of biofilm growth and metabolite dynamics. Some of the frequently applied devices are microtiter plates, Calgary biofilm device, Biofilm Ring Test, Robbins device and the drip flow biofilm reactor. Additionally, three types of rotary biofilm reactors such as the rotary disk reactor, the rotary annular reactor and the concentric cylinder reactor have been applied [1] and novel designs are continuously being introduced [2].

**Practical Applications**

The presented device can be applied for quick assessment of biofilm formation in (1) process industry where different piping surfaces are being tested for biofouling; (2) shipping industry where various anti‐biofouling paints applied on marine structures are being examined for biofilm prevention; (3) biotechnology industry where bioproduct (e.g., enzymes, antibiotics, and pigments) formation is associated with biofilm formation, that is, productive biofilm concept; (4) medical applications where the effect of various medications are evaluated for biofilm formation on medical implant surfaces; and (5) environmental applications where biofilm formation is correlated with enhanced wastewater treatment process efficiency and investigations on biofilm mediated remediation of aquatic organic pollutants as well as sequestration of heavy metals.

A novel biofilm cultivation device termed “Enhanced Surface Area Conico Cylindrical Flask” (ES‐CCF) was applied successfully for the cultivation of single and multispecies biofilms [3, 4, 5, 6, 7, 8, 9, 10, 11]. The device was developed by Jadavpur University, India and patented by the Council of Scientific and Industrial Research, India [12]. The novel ES‐CCF provides over 80% more surface area for biofilm attachment and growth compared to a similarly sized Erlenmeyer flask. Furthermore, it offers the flexibility to modify the growth surface's characteristics, presenting options between hydrophilic and hydrophobic properties. The ES‐CCF additionally offers external aeration similar to that of a bioreactor, thereby enhancing its range of potential applications. The vessel can be placed in standard rotary shaking platforms when the influence of shear forces on biofilm formation needs to be investigated.

The ES‐CCF, however, has certain limitations. If a slide is removed during an ongoing experiment for analysis or observation, it cannot be placed back again. This Technical Report describes an improvement to overcome this drawback and modification of the ES‐CCF to ensure the easy introduction of substances and removal of contents from the ES‐CCF for observation or sampling or further experimentation. This modification guarantees the least distortion of the biofilm when the sample is taken out for observation or studies where the morphology must remain intact. We named the modified ES‐CCF lacking the conical part as an enhanced surface cylindrical flask (ESCF). A popular three‐dimensional (3D) printing technology, fused deposition modeling (FDM) utilizes a variety of thermoplastic filaments, including biodegradable polylactic acid (PLA) [13, 14], and this method was applied to print the ESCF. Growing consumer knowledge of the adverse environmental effect of petrochemical‐based polymers and a worldwide transition away from conventional plastics has resulted in greater interest in bio‐based polymers. Hence, biodegradability of the 3D print material was an important consideration for selection. Additionally, the current bio‐based polymers offer significantly improved technical specifications than their predecessors. PLA is well known as a biodegradable thermoplastic source for use in 3D printing by the FDM method. The glass transition temperature of PLA is 55°C, the melting point (*T*_m_) is 180°C, and this biopolymer has a wide temperature range for operation. Moreover, the polymer has good biocompatibility and high strength. PLA is widely used in 3D printing because it is easy to print. Printing with a heated bed makes the experience much satisfying and 70°C heated bed is sufficient to achieve high‐quality print. Thus, PLA is the best material of choice for beginners [15]. Although generally believed that PLA‐derived products, for example, the ESCF is not autoclavable, it was demonstrated that 3D printing applying FDM technology produced PLA‐constituted products that were steam sterilisable. Thirty‐one nosocomial pathogens were eliminated at 134°C sterilization temperature and 2.1 bar pressure [16]. In this Technical Report, we explain how the ESCF vessel was 3D printed in its entirety and applied for biofilm growth and visualization by confocal laser scanning microscopy (CLSM). We believe this is the first example of an intricately designed and versatile biofilm‐cultivation device to be produced by additive manufacturing (3D printing) at the laboratory scale.

## 2 Materials and Methods

### 2.1 Design of the ESCF

**FIGURE 1:**
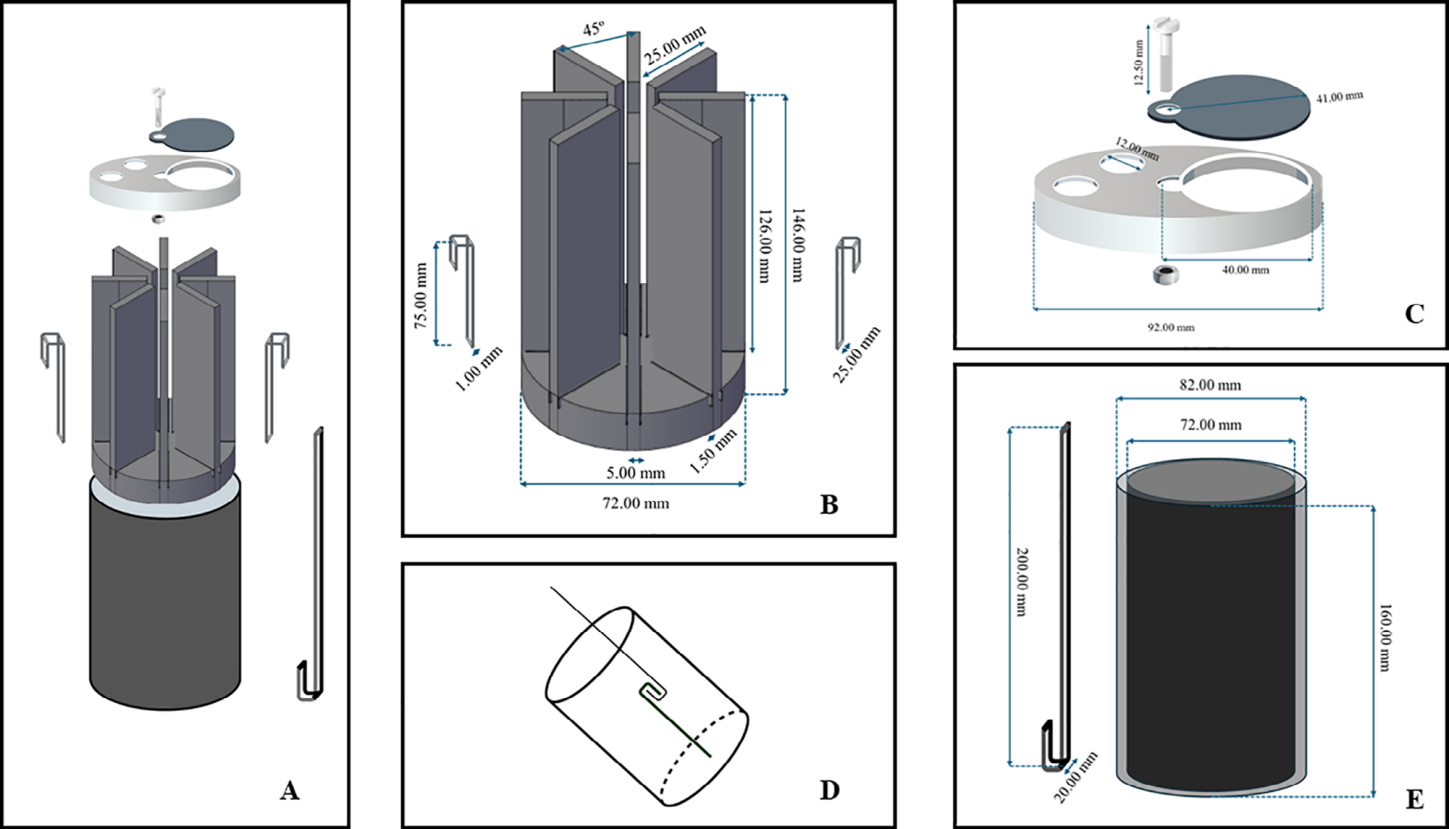
(A) Exploded view of the enhanced surface conical flask (ESCF) showing the different components (from top to bottom): window‐flap mechanism, internal assembly, slides and spatula, cylinder. (B) Surface area enhancing internal assembly with slides. (C) Detailed view of window‐flap mechanism. (D) Interlocking mechanism for withdrawal of slides from the internal assembly with the spatula. (E) Cylinder and spatula.

The ESCF comprised of an internal chamber consisting of rectangular strip(s) embedded in a raised platform having slots by the side of the rectangular strips. Specially designed slides could be inserted within the slots provided. As another improvement of our previously designed vessel [12], we introduced a removable and rotatable flap fitted in the cap that uncovered a cut‐out window area within the cap above a pre‐determined slide, and the required slide could be taken out from its respective slot with a designed spatula. The cells and substances on the pulled‐out slide after completion of any observation/experimentation could be placed back to its previous position inside the slot with the help of the spatula. This removable and rotatable flap when rotated to expose the cut‐out window allowed fluids and solids to be placed into or taken out of the flask. Sampling of fluid and cells from inside the flask could be carried out without completely disengaging the components of the vessel. Two holes present on the vessel top also served as ports through which the degree of aeration into the flask could be controlled. Additionally, the rotatable flap along with the two holes served as points for placing and removing of probes and electrodes. Vessel design and configuration art were patented by Jadavpur University, India [17] and depicted in Figure 1 and Video S1.

### 2.2 Computer Aided Design and 3D Printing of ESCF

At first, the thermoplastic filament was loaded into the FDM printer (Stratasys Ltd. Model No. F170, USA). Next, a digital 3D model was created using computer aided design (CAD) software which was sliced into thin horizontal layers using Grab CAD slicing software. Subsequently, FDM printer's extruder head heated the thermoplastic filament to its melting point, turning it into a semi‐liquid state. The melted filament was then extruded through a nozzle onto the build platform in the pattern determined by the sliced layers. The nozzle moved in the X and Y directions, while the build platform moved downwards in Z direction after each set of depositions in XY plane. The process repeated layer‐by‐layer, with each new layer bonding to the previous one as it cooled and solidified. This successive layering created the final 3D object. The nozzle temperature was 190°C, bed temperature 70°C, raster angle ±45°, print speed 50 mm/s, degree of filling 50% and layer thickness 0.254 mm. Crisscross orthogonal raster deposition was considered as infill pattern, while build orientation regarded each component to be fabricated according to its design specification.

### 2.3 Biofilm Cultivation in the ESCF

Modification of a standardized method [8] was followed for biofilm cultivation in the ESCF. Vessel components were disassembled and immersed in 50% (v/v) benzalkonium chloride overnight for sterilization [18]. Components were washed thoroughly in ultrapure type‐II distilled water, dried at room temperature, and further UV‐sterilized (Lamp model TUV15W/G15T8, Philips, The Netherlands) in a laminar airflow bench for 1 h [9]. PLA slides were inserted aseptically inside the slots of the eight‐strip inner arrangement of the ESCF. Biofilm induction experiments were performed with pure cultures of four bacteria: *Klebsiella pneumoniae* (MTCC 618), *Pseudomonas aeruginosa* (MTCC 424), *Staphylococcus aureus* (MTCC 2940), and *Escherichia coli* (MTCC 1195). Each bacterium was cultivated in the ESCF in 150 mL tryptone soy broth supplemented with 1% glucose [19]. Five‐milliliter culture of each bacterium in nutrient broth (OD_600_: 0.8) was used as the inoculum [20], and cultures were incubated at 37°C. Biofilm growth was observed through the movable‐flap aperture of the ESCF and CLSM of the biofilm was performed after 72 h of incubation.

### 2.4 Visualization of Biofilms Cultivated in the ESCF

Three slides were removed aseptically from each batch culture after 72 h, air dried, and fixed with methanol‐Carnoy's fixative (methanol:chloroform:acetic acid − 6:3:1) before washing with 1 mL phosphate buffered saline (PBS: 1X concentration) [21]. Modification of previous methods [7, 8] was followed to visualize the biofilms developed in the 3D‐printed ESCF by CLSM. Slides were treated with calcein AM solution [22] for staining the EPS component of the biofilms. Subsequently, propidium iodide (PI) staining was performed for visualization of the biofilm cells [23]. Slides were washed with 1 mL of PBS and 1 mL of tris(hydroxymethyl) aminomethane (TRIS) buffer. Each bacterial biofilm was observed and imaged using Zeiss confocal laser scanning microscope (Carl Zeiss Model No. LSM880) equipped with 63X oil‐immersion objective lens and analyzed with ZEN Blue software version 3.7. Red fluorescence of PI stain was visualized at excitation 494 nm and emission 517 nm while green fluorescence of calcein AM stain was observed at excitation 535 nm and emission 615 nm. 2D images thus obtained were converted to 3D surface plots using the Deep‐Z plugin installed within the Fiji/ImageJ package [24, 25].

## 3 Results and Discussion

**FIGURE 2:**
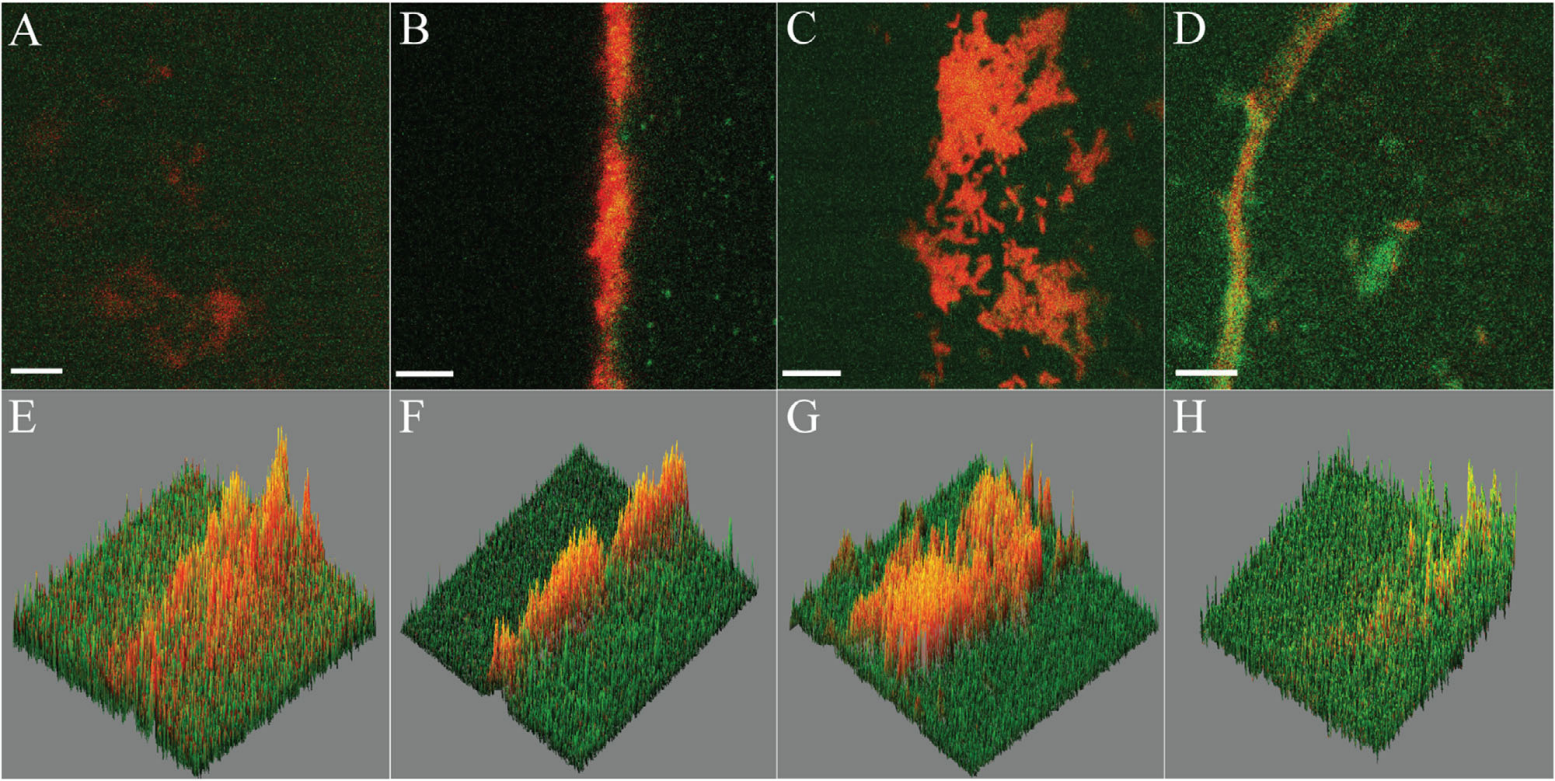
Upper panel: Confocal laser scanning microscopy (CLSM) 2D images of (A) *Staphylococcus aureus* (MTCC 2940), (B) *Klebsiella pneumoniae* (MTCC 618), (C) *Pseudomonas aeruginosa* (MTCC 424), and (D) *Escherichia coli* (MTCC 1195) cultivated in the enhanced surface conical flask (ESCF) for 72 h after inoculation at 37°C. Green fluorescence of calcein AM (excitation maxima ∼494 nm, emission maxima ∼517 nm) and red fluorescence of propidium iodide (excitation maxima ∼535 nm, emission maxima ∼615 nm) were recorded. Scale bar size of each image is 40 µm. Lower panel: Three‐dimensional surface plots of the aforementioned bacterial biofilms developed in the ESCF and visualized through CLSM. (E) *S. aureus* (MTCC 2940), (F) *K. pneumoniae* (MTCC 618), (G) *Pseudomonas aeruginosa* (MTCC 424), and (H) *E. coli* (MTCC 1195).

3D printing was selected over conventional manufacturing methods due to its capability to produce intricate geometries with greater precision. The holder (Figure 1) comprised of eight long rectangular pillars arranged in a circular fashion, each maintaining 1 mm gap with its base to accommodate slides on each face. Successful printing of this complex geometry would ensure precise fitting within the cylindrical flask. The 3D‐printed flask wall had 5 mm thickness, providing ample strength while the flask cap was designed and fabricated to prevent contamination during bacterial growth. Slides were fabricated to facilitate convenient analysis of bacterial growth under both light microscopic and laser scanning methodologies. FDM printing was able to create all the complex geometries with PLA as the material of construction that proved instrumental in the overall fabrication procedure. For the successful assembly of all components, it was essential that the geometry specified in the CAD model was accurately fabricated. This precision was critical for ensuring optimal functionality of the components during operational phases. Geometric precision was assessed and is detailed in Table 1. The associated figure illustrates various key component assemblies necessary for the product's functional performance. To enhance dimensional accuracy, 0.254 mm layer thickness was selected. This thickness allowed effective diffusion between successive layers during deposition, leading to improved surface quality and adequate dimensional precision. CLSM imaging revealed that slide surfaces were completely covered by biofilm exopolymeric substances (EPS, visible as green when stained with calcein AM) while bacterial cells appeared as red (stained with PI). CLSM images indicated identical EPS formation for the four test bacteria; although the amounts of cells embedded in the EPS matrix were different (Figure 2).

**TABLE 1:** Geometric precision achieved during the 3D printing of enhanced surface conical flask (ESCF) with associated figure illustrating various key component assemblies.

Components	Designed dimensions (mm)	Measured dimensions (mm)	
		Mean (mm)	Standard deviation (mm)
Flask inner diameter (A)	72	72.606	0.201
Slide holder diameter (B)	72	72.214	0.069
Rectangular column thickness (C)	5	5.082	0.035
Gap for slide fitting (D)	1.5	1.502	0.021
Slide thickness (E)	1	1.14	0.042
Cap inner diameter (F)	82	82.09	0.063
Flask outer diameter (G)	82	82.032	0.033
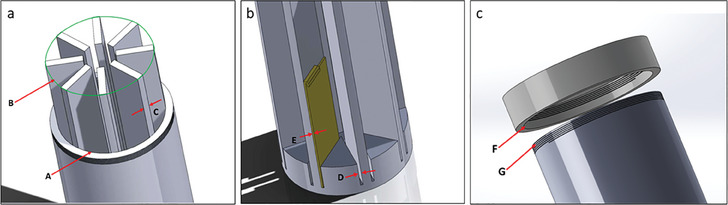			

The flexibility of 3D printing supports optimization of geometries and hence finds increasing applications in the fabrication of biotechnological devices [26]. A comparison of our design with the currently available models produced by 3D printing is discussed now. The FlexiPeg biofilm device produced by standard 3D printing [27] comprised of a lid having pegs that were implanted through a silicone mat to fit into a 96‐well plate. Application of the FlexiPeg with various peg materials and coatings was validated by growing biofilms of *E. coli* and *K. pneumoniae*. A flow cell for microscopy was designed and 3D‐printed [28] that provided shear‐controlled flow and accommodated samples of different geometry. The authors grew dental biofilms in situ on custom‐made glass slabs and established the significant influence of salivary flow on pH changes inside the biofilms. Another biofilm flow system (Duckworth Biofilm Device [DBD]) was presented [29] with the aim of better simulation of a chronic infected and exuding wound. The device possessed series of wells for growing 12 biofilms across four separate channels. Triplicate biofilms could be cultured and sampled during experiments without disturbing continuing biofilm growth.

In comparison to the 3D‐printed biofilm cultivation devices currently available, the ESCF presented several advantages. For example, our designed vessel possessed much advanced features when compared to the peg biofilm device [27]. Additionally, the introduction of shear forces was not feasible in the peg biofilm device. On the other hand, the ESCF may be placed on standard rotary shaking platforms to generate shear within the fluid surrounding the biofilm if such shear forces were desired by the experimenter. Shear forces affect the strength of attachment and the structure of a biofilm. CLSM was not done on the biofilms developed in the peg biofilm device [27] nor the microfluidic devices [28] and DBD [29]. CLSM is the bedrock of microbial visualization of biofilms [30] and such analysis can be easily done with biofilms cultivated in the ESCF. The comparable devices were designed for very specific applications on dental and wound biofilms, respectively [28, 29]. Thus, they required complicated experimental steps such as placing the glass slab inside the flow cell without touching the superficial biofilm, as well as sealing of the flow cell with the coverslip [28]. The placement of the disc of noble agar to rest on the support ledge and the further insertion of a cellulose membrane on the top of the noble agar disc [29] was intricate too. On the other hand, the placement and removal of slides in the ESCF was simpler in comparison to these two previously described models. Yet another advantage of the ESCF over the available models was the possibility of the introduction of electrodes into the liquid surrounding the biofilm for constant monitoring of pH or dissolved oxygen. Similarly, anoxic conditions may be created by the flow of gases such as nitrogen or carbon dioxide, which was not feasible in the previous models described [27, 28, 29].

## 4 Concluding Remarks

We designed and 3D printed a biofilm‐cultivation device that allowed easy insertion and removal of microscopic slides for CLSM. Advantageously, the ESCF fitted easily in standard rotary shaker platforms, permitted measurements of pH, dissolved gases and metabolites in the liquid surrounding the biofilm as well as creation of anoxic environment inside the vessel. Four commonly investigated microorganisms were successfully cultivated on microscopic slides inside the ESCF vessel and visualized through CLSM. The presented device would be useful for quick assessment of biofilms on a variety of growth surfaces under different culture environments and thus should find widespread application in the studies on industrial, medical, and environmental biofilms.

## Supporting information



Supporting Information

## Data Availability

All data are provided in the article.
